# Forecasting Social Unrest Using Activity Cascades

**DOI:** 10.1371/journal.pone.0128879

**Published:** 2015-06-19

**Authors:** Jose Cadena, Gizem Korkmaz, Chris J. Kuhlman, Achla Marathe, Naren Ramakrishnan, Anil Vullikanti

**Affiliations:** 1 Virginia Bioinformatics Institute, Virginia Tech, Blacksburg, VA, USA; 2 Department of Computer Science, Virginia Tech, Blacksburg, VA, USA; 3 Department of Agricultural and Applied Economics, Virginia Tech, Blacksburg, VA, USA; University of Warwick, UNITED KINGDOM

## Abstract

Social unrest is endemic in many societies, and recent news has drawn attention to happenings in Latin America, the Middle East, and Eastern Europe. Civilian populations mobilize, sometimes spontaneously and sometimes in an organized manner, to raise awareness of key issues or to demand changes in governing or other organizational structures. It is of key interest to social scientists and policy makers to forecast civil unrest using indicators observed on media such as Twitter, news, and blogs. We present an event forecasting model using a notion of activity cascades in Twitter (proposed by Gonzalez-Bailon et al., 2011) to predict the occurrence of protests in three countries of Latin America: Brazil, Mexico, and Venezuela. The basic assumption is that the emergence of a suitably detected activity cascade is a precursor or a surrogate to a real protest event that will happen “on the ground.” Our model supports the theoretical characterization of large cascades using spectral properties and uses properties of detected cascades to forecast events. Experimental results on many datasets, including the recent June 2013 protests in Brazil, demonstrate the effectiveness of our approach.

## 1 Introduction

Social media has become a window into happenings on the ground, from earthquakes [1] to specific news stories [2]. A key population-level event is civil unrest, i.e., protests, strikes, and occupy events, wherein civilian populations mobilize to raise awareness of key issues. As is evident from recent protests in many countries (e.g., Egypt, Turkey, Brazil), social media plays an important role in documenting, triggering, mobilizing, or even quelling such events, e.g., see [3, 4]. However, it is not clear when preliminary chatter observed on social media becomes a true precursor for a protest, and thus understanding the structure of the tweet behavior is a relevant problem.

While Twitter is used for many purposes (e.g., discontent expression, event reporting, planned protest recruitment), all of which can be used in a predictive model for civil unrest, we focus on modeling activity cascades (using a formulation of [5–7]) as a uniform precursor for civil unrest forecasting. Our goal is to design a model that forecasts the date of the event. Cascades, as is well known, help formalize the spread of influence and information, e.g., see [8–12]. Informally, cascades are subgraphs (often trees) that capture the spread of influence from a node to its newly activated/influenced neighbors and descendants.

There are many notions of cascades, which afford varying levels of formal characterization and utility. For instance, in Bakshy et al. [8], an edge *A* → *B* is included in a cascade only if it can be argued that some action (e.g., a posting or use of a URL) by *B* can be directly attributed to *A*, which makes the analysis intensive in terms of data and computation; also, the mathematical analysis under their model becomes challenging. Another common approach is to define cascades in terms of the (random) subgraph over which diffusion processes like linear threshold and independent cascades models spread, e.g. [12, 13]. Here, we use a simpler notion of cascades (referred to as “activity cascades”), introduced by [5–7]. Informally, such a cascade consists of a tweet emitted by a user *u*, and the tweets of the users who see/mention *u*’s message (for instance, her direct followers or users who mention her) given that those tweets are sent within a small time interval (denoted by Δ), and so on (see Section 3.1 for a precise definition). This turns out to be a special case of Hawkes processes [14–16], which are based on mixtures of mutually exciting point processes. Although simpler to define, we demonstrate that this notion of cascades has good predictive power for modeling civil unrest, and is also amenable to rigorous analysis. Our key contributions are focused on the following three questions:

**1. When do large activity cascades happen?** A common empirical observation is that cascades seldom become very large. We rigorously prove necessary and sufficient conditions for large cascades in terms of spectral properties of the underlying graph (Section 3.2); these also imply a similar characterization for a class of Hawkes processes. We find that this characterization closely matches our empirical observations for synthetic traces. Specifically, our analyses show if the spectral radius of a cascade graph (defined in Section 3.2) is below a particular constant, then large cascades are not possible. Our techniques build on approaches for analyzing the spread of epidemics [17–19], and are the **first such results** for cascades of this kind.

**2. Are there critical subsets of users that contribute to cascades?** We study the questions of identifying critical subsets of users responsible for formation and survival of cascades, and formalize these as two complementary problems: CriticalSetFormation (CSFP) and CriticalSetShattering (CSSP). We show both to be NP-complete, and we evaluate different greedy heuristics to approximate them empirically by studying large, monthly cascades for all (country, month) combinations for Brazil, Mexico, and Venezuela over a 15-month period. Our results for CSSP show that a very small set of users are critical for a cascade to exist—their non-participation causes the cascade to shatter. We also prove that a high degree strategy gives a constant factor approximation for CSSP in random power law graphs. On the other hand, the results for CSFP suggest that unless a large fraction of users participate a cascade cannot exist. Thus, one needs a reasonable fraction of users, plus some critical users for a cascade to exist. These are validated through empirical observations in Section 4.2.

**3. Can we forecast protests using activity cascades?** Since large cascades are not very common, their occurrence signals a big event. We analyze over 353 million tweets from three Latin American countries over a 1.5-year period, and consider activity cascades formed by a filtered set of tweets. These tweets contain at least 3 keywords from a dictionary that has over 900 words in English, Spanish and Portuguese, related to civil unrest activities. This ensures that the resulting activity cascades will be relevant to the topic of civil unrest. Next, we build a feature set based on the structural properties of the cascades, to be used as predictors of social unrest. Statistical models are then used to remove redundancies among features and make predictions of events from the reduced feature set. The model is tested on multiple countries for robustness and compared against a baseline model. We show that our approach can ‘beat the news,’ i.e., contribute a lead time of one to two days over the reporting of a protest in major news media, with an accuracy of over 0.75. It can even predict black swan events like the Brazilian Spring with an accuracy of 0.83. (Section 4.6).

Our paper helps explain the model and observations of [5–7] rigorously, especially conditions for occurrence of large cascades. Since their frequency is relatively low, their occurrence is a signal of significant events—-this corroborates with the observations of [5], and is the basis of our approach for forecasting protest events.

### 1.1 Related work

Analyzing traffic trends in twitter and other social media sites is a very active topic of research. Some of the specific applications include identifying specific news stories [2, 20], tracking natural disasters [1], predicting stock market moves [21, 22] and understanding political or cultural events [5, 6, 23, 24]. Yang et al. [25] predict temporal patterns in the usage of specific hashtags in social media data. Hutto et al. [26] show that increases in followers on Twitter are predicated on social behavior, message content, and social network structure variables in roughly equal proportions. Hsieh et. al. [27] demonstrated that experts could not match the crowd in identifying future interesting news stories. Most of these works have focused on counts of keywords and hashtags, and do not capture peer influence in the use of such terms. Peer influence is often modeled by diffusion processes, such as linear threshold and SI/SIS/SIR epidemic models, e.g., [12, 13]; in this context, cascades are used to refer to the (random) subgraph on which the diffusion spreads. There has also been a lot of work on using semantic information for attributing influence more carefully, e.g., [8–11], as discussed in Section 1.

A simpler notion of cascades is studied by [5, 6] in Twitter follower graphs and by [7] in the mentions graphs. Large cascades involving protest-related hashtags are found to occur infrequently. Our formulations extend point process models, which have been studied extensively. Two closely related approaches are by [15, 16]. Simma et al. [15] consider a model in which a Poisson process triggers other Poisson processes. They develop an EM algorithm to infer the random forest of events, which captures the cause of each event. Zhou et al. [16] use a multi-dimensional Hawkes process.

There are other works that utilize models for characterization and prediction. Linear regression models using average tweet rates, and tweet rate time series (per-day tweet rates over a 7-day period), have been used to predict box-office revenues from movies [28]. A classifier and hidden Markov model have been used with tweet content to establish the onset and end of identified events (versus event prediction) [29]. Natural language processing and LDA have been used to identify topics that capture collections of events identified in tweets; a linear regression model is then used to predict crimes [30].

With respect to forecasting social unrest, [31] provides empirical data showing that increases in food prices correlate with protests in 2008 and 2011. Rather than predicting specific unrest events, [32] uses a 2-parameter dynamics model to predict the distributions of numbers of unrest events per year, for many regions of the world. Disease outbreaks, deaths, and riots are forecasted with topic detection and tracking using news articles, and a Bayes scheme to compute the probability of some event, given other events occurring beforehand [33]. A tension parameter, based on hashtag usage, was shown to correlate well with clashes in Egypt between secularists and Islamists [34]. A generalized least squares model of political *violence* [35] is used to predict the overall level of violent activity in a country, by year. By contrast, we are interested in violent and non-violent protests.

Network characteristics and spectral bounds have been used for analyzing epidemic spread in networks. Ganesh et al. [17] develop necessary and sufficient conditions for the duration of an SIS process; our analysis strongly builds on this approach, but our model requires the use of a variant of the node expansion, instead of edge expansion. Similar spectral radius bounds are also considered in [18, 19] for the SIS process.

Our work can be differentiated from the above studies in the following ways. This is the first work of which we are aware that predicts daily civil unrest events in multiple countries using a combination of different graph cascade characteristics. Further, we explain theoretically and demonstrate empirically conditions that delineate small and large cascade regimes, using spectral properties of the underlying graphs.

## 2 Materials

### 2.1 Twitter Dataset

For event prediction, we use a set of over 353 million tweets collected for Brazil, Mexico and Venezuela for the period of May 2012 through November 2013. Our dataset constitutes a 10% sample of the tweets for these countries during the above time period.

Our analysis is done separately for each country, and, as a pre-processing step, the tweets are filtered by country (using geolocation codes, place identifiers, language detection, author identification, and other enrichment processes), ignoring tweets for which a country of origin could not be determined. Next, we organized a vocabulary of 614 protest-related words (such as march, riot, strike, organize, democracia, conflicto, revolucion, criminalidade), 192 keyphrases (such as “right to work”, “marcha por la paz”), and 105 country-specific key players (which include important public figures, political parties, labor unions), collectively referred to henceforth as keywords. Compiled by social scientists who are experts in the region, the keywords include English, Spanish, and Portuguese translations. We then subselect tweets which contain at least 3 keywords from our vocabulary. Tweet volumes before and after filtering are shown in Table 1.

**Table 1:** Number of tweets for the period May 2012 to Nov 2013.

**Country**	**Raw**	**Filtered**
Mexico	97,873,616	3,524,695
Venezuela	105,938,438	6,683,834
Brazil	150,147,141	1,575,041

### 2.2 Follower Data

We obtained the follower network for a subset of the users who appear as authors in our dataset, for each country by using Twitter API from a large number of machines. The size of the graphs for the three countries are the following: Mexico has 79,598 nodes and 1,437,687 edges, Venezuela has 312,241 nodes and 21,119,120 edges, and Brazil has 142,176 nodes and 6,854,368 edges.

### 2.3 Gold Standard Report (GSR) Datasets

GSR datasets are compiled by an independent group, selected by IARPA (Intelligence Advanced Research Projects Activity), which is comprised of social scientists and experts on Latin America. A small set of well-reputed newspapers for each country are used to identify the instances of civil unrest events to be included in the GSR. For each event, the GSR captures the when, where, who and why of the event, i.e. the date of the event, its geographic location, the population protesting (e.g. labor, medical workers, general population) and the reason for the protest (i.e., the event type, e.g., economic, political, resource). Here we are interested in forecasting primarily the if/when of the event (although text classification and geo-coding of the tweets reveals insight into where/who/why, something we do not study here further).

## 3 Methods

### 3.1 Activity Cascades

Let *G* = (*V*, *E*) denote a directed graph, with *N*_*o*_(*u*) and *N*_*in*_(*u*) denoting the set of out-neighbors and in-neighbors for a node *u* ∈ *V*, respectively. The nodes represent Twitter users. We consider two kinds of graphs—a *follower* graph and a combined *mentions* and *retweet* graph. An edge (*u*, *v*) has different interpretations depending on the graph, as discussed below. We assume each user *u* sends at most one tweet at a time (with one second granularity), so a node-time pair (*u*, *t*) identifies a tweet. For a node *u* ∈ *V*, time *t* and time interval Δ, we define a cascade *C*(*u*, *t*, Δ) to be a set of tweets/node-time pairs in the following recursive manner, using the formulation of [5–7].

- If there is no tweet *driven by* node *u* at time *t*, then *C*(*u*, *t*, Δ) = *ϕ*.
- Else, *C*(*u*, *t*, Δ) = {(*u*, *t*)}∪{*x* ∈ *C*(*v*, *t*′, Δ):*v* ∈ *N*_*o*_(*u*), *t*′ ∈ (*t*, *t*+Δ]}

The term *driven by* will be explained below. This general notion of cascade makes no prior assumptions about the nature of the edges connecting the nodes of the graph, which gives us the flexibility to define the neighborhood of a node in different ways. Though this definition does not explicitly look for any correlation between the messages of *u* and *v* (which is used by, e.g., [8–12]), we use it on a set of tweets that are already filtered for protest related keywords, as done by [5–7], which brings in some correlation. Also, note that this notion of cascades is different from the more commonly studied notion associated with diffusion processes, e.g., [12, 13]—here, a cascade is a random subgraph on which the influence spreads.

In this paper, we study two types of activity cascades: *follower* (F) and combined *mention* plus *retweet* (MRT) cascades, defined, respectively, by the *follower* and *mention* and *retweet* graphs, each of which models a different kind of interaction between users in the Twitter network. Our methodology is the same as [36], except that we also include retweets.

**Fig 1:**
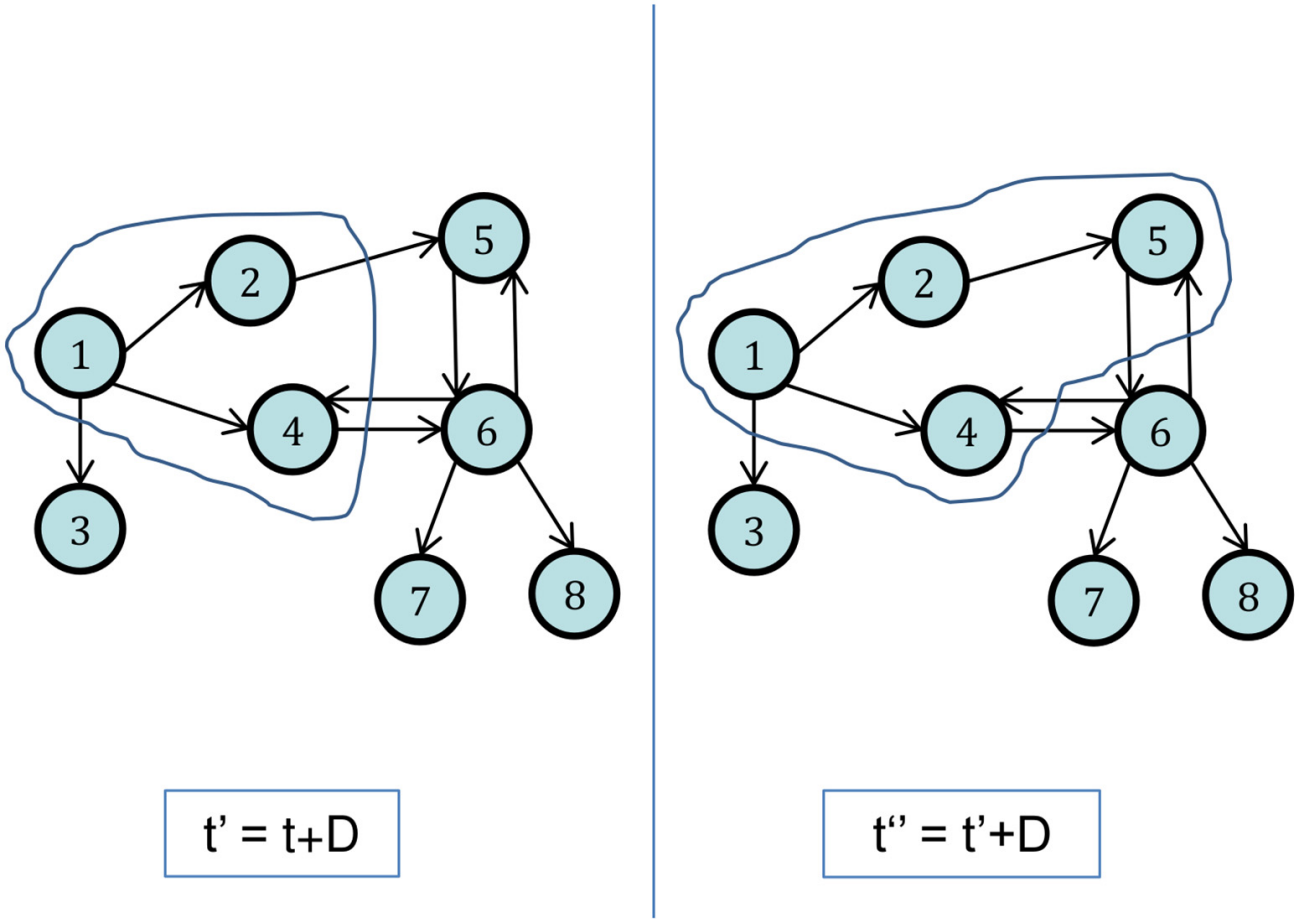
Formation of cascades in the Twitter follower network. At time *t*, node 1 posts a tweet. Nodes 2 and 4 post at times *t*_2_ and *t*_4_ between *t* and *t*′ = *t* + *D*. Node 5, which follows 2, posts at some time *t*_5_ between *t*′ and *t*″ = *t*′ + *D*. Therefore, the cascade *C*(1, *t*, *D*) is *C*(1, *t*, *D*) = {(1, *t*), (2, *t*_2_), (4, *t*_4_), (5, *t*_5_)}.

In a follower graph, for every node *u* ∈ *G*, *N*_*o*_(*u*) is the set of Twitter followers of *u*, and *N*_*in*_(*u*) is the set of Twitter friends of *u* (i.e. users followed by *u*). From this definition, follower cascades in Twitter emerge in the following manner: a user *u* posts a tweet at time *t* starting a cascade where she is the only participant. For each follower *v* of *u* who posts a tweet at some time *t*′ ∈ (*t*, *t*+Δ], (*v*, *t*′) is added to this cascade, and so on, as illustrated in Fig 1. This process is repeated until no more users can be added to the cascade.

We combine mentions and retweets to form an MRT graph because both types of tweets indicate influence between pairs of users. Suppose a user *w* with name *W* composes a tweet at time *t*_1_ that mentions another user *u* with name *U*, where *W* and *U* are sequences of characters of the form [*a*−*zA* − *z*0 − 9_]+. In the following, we specify the concatenation of two sequences of characters, *A* = (*a*_1_, *a*_2_, …, *a*_*q*_) and *B* = (*b*_1_, *b*_2_, …, *b*_*r*_), using the operator ⊕, as *A*⊕*B* = (*a*_1_, *a*_2_, …, *a*_*q*_, *b*_1_, *b*_2_, …, *b*_*r*_). Let *P*_*W*_ be the payload or content of the tweet of *W*; *P*_*W*_ is a sequence of characters. Because *w* mentions *u*, we have (@)⊕*U* ⊆ *P*_*W*_, which produces the directed edge (*u*, *w*) of the MRT graph. This edge has the semantics that *u* influences *w*. Analogously, if *x* with name *X* retweets a message from user *w* at time *t*_2_, where (*RT* @)⊕*W* ⊆ *P*_*X*_, then we have the directed edge (*w*, *x*), where the semantics are the same as in the mentions edge: *w* influences *x*. If the two tweets occur such that *t*_2_ ∈ (*t*_1_, *t*_1_+Δ], then the two edges link up to form a directed path of length 3, *u* → *w* → *x*, and the cascade *C*(*u*, *t*_1_, Δ) = {(*u*, *t*_1_), (*w*, *t*_2_)}.

We have several notes. The term *driven by* indicates the user that instigates the cascade. For a follower graph, the instigator is the user that sends the first tweet of a cascade. For an MRT graph, the instigator is the first influencer of a cascade. Second, users (nodes) in an MRT graph with zero out-degree (*x* in this example) are not included in the MRT cascade because there is no evidence that these nodes influence other users. Also, a single tweet can produce multiple edges in an MRT graph. Since retweets of an original tweet preserve the original tweeter, no matter how many times the original tweet is (sequentially) retweeted, a set of these retweets (without mentions) produces a star subgraph of the MRT graph. Finally, MRT cascades, unlike follower cascades, directly use tweet payloads; however, F cascades utilize the follower graph.

We provide additional definitions that will be useful in forecasting social unrest. We say that a (follower or mentions/retweet) cascade *C* is *active* on day *d* if there exists at least one message or tweet (*u*, *t*) by user *u* at time *t*, such that *t* is some time during day *d* and (*u*, *t*) is an element of *C*. The *size* of a cascade is the number of tweets comprising it. A *user* or *participant* is a tweeter.

### 3.2 Characterizing large cascades in terms of graph properties

Our empirical results suggest that large and long cascades are rare, and arise within communities of users. We now attempt to explain this behavior by relating it to the spectral properties of the graph, by considering a formulation based on a slight relaxation of the notion of cascades: We consider a cascade starting at a random initial node *u*_0_ at time *t*_0_; (i) *X*(0) denotes the initial configuration. We say that (*u*_0_, *t*_0_) is active in the cascade at time *t*_0_. Following our definition, we will think of a cascade as consisting of tweets indexed by user-time pairs (*u*, *t*); (ii) The number of tweets sent by each user *u* is a Poisson process with parameter *α*_*u*_; (iii) If user *u* sends a message at time *t*, and some other user *v* with *u* ∈ *N*_*o*_(*v*) is active at time *t*, then (*u*, *t*) becomes active in the cascade at time *t*; (iv) A tweet (*u*, *t*) ceases to be active after a (random) time duration *D*(*u*, *t*) drawn from an exponential distribution with parameter *δ* = 1/Δ; and (v) The cascade *C*(*u*_0_, *t*_0_) dies when there are no more active tweets in it.

We now model this as a Markov process **X(t)** with values in ℕ^*V*^. Let *X*_*u*_(*t*) denote the number of messages by user *u* that are active at time *t*. Then, the cascade evolves in the following manner:

$$\begin{array}{cc} X_{u}:\;\text{increases}\;\text{by}\;\text{1} & \text{at}\;\text{rate}\;\alpha (u)\;\text{if}\;\{v\in N_{in}\left(u\right):X_{v}>0\}\neq \phi \end{array}$$ (1)

$$\begin{array}{cc} X_{u}:\;\text{decreases}\;\text{by}\;\text{1} & \text{at}\;\text{rate}\;\delta X_{u} \end{array}$$ (2)

Every cascade eventually becomes inactive, since *X*_*u*_ = 0 for all *u* is the unique absorbing state for this Markov process. The *lifetime* of the cascade, the duration for which it lasts, is precisely *T* = sup{*t*:*X*_*u*_(*t*) > 0,for some *u* ∈ *V*}. We now derive necessary and sufficient conditions for obtaining large cascades.

#### 3.2.1 Multivariate Hawkes Processes

Our formulation above makes it a special case of the multivariate Hawkes processes, as we now discuss. A Hawkes process **N_t_** is a type of self-exciting counting process characterized by a time-dependent intensity (rate) *λ*(*t*) [14–16].

Let **N_d_(t)** be a multidimensional counting process, where *d* ∈ {1, …, *D*} denotes a dimension (with *D* being the number of dimensions). Let *λ*_*d*_(*t*) denote the intensity of *N*_*d*_(*t*). The process is defined in the following manner:

$$\begin{array}{c} \lambda_{d}\left(t\right)=\mu_{d}\left(t\right)+\sum_{d^{\prime }=1}^{D}\int_{-\infty }^{t}\kappa_{d^{\prime }d}\left(t-s\right)dN_{d^{\prime }}\left(s\right), \end{array}$$

where *μ*_*d*_ is a base intensity for dimension *d*, and *κ*_*d*′*d*_(*τ*) is a kernel function describing the influence of the previous events in dimension *d*′ on the current rate on *d*.

For our formulation, let each node *u* ∈ *V* be a separate dimension, and let **N_u_(t)** be the number of messages contributed to an ongoing cascade. We have *μ*_*u*_(*t*) = 0 and the kernel function as *κ*_*vu*_(*τ*) = *α*_*vu*_ × *κ*(*τ*), where: (i) *α*_*vu*_ = *α*_*u*_ *N*_*v*_(*t*) if *v* ∈ *N*_*in*_(*u*); otherwise, *α*_*vu*_ = 0. Here, *α*_*u*_ is the (fixed) tweeting rate of *u*, and *α*_*vu*_ describes the fact that *u*’s contributions to the cascade are proportional to her in-neighbors’ contributions (i.e. her friends in the follower graph); and (ii) *κ*(*τ*) = 1 if 0 < *τ* ≤ Δ; otherwise *κ*(*τ*) = 0. As a result, we have:

$$\begin{array}{c} \lambda_{u}\left(t\right)=\alpha \left(u\right)\sum_{v\in N_{in}\left(u\right)}\left(N_{v}\left(t\right)-N_{v}\left(t-\Delta \right)\right) \end{array}$$

We use the process **X(t)** below for our discussion, since it simplifies the analysis; our results hold for a class of Hawkes processes with the kind of kernel function mentioned above.

#### 3.2.2 Conditions for Small Cascades

We now derive conditions when the maximum cascade size is *O*(log*n*), with high probability, where *n* is the number of nodes. The process **X(t)** is non-linear, making it quite complex to analyze; instead we consider the following relaxation **Y(t)**:

$$\begin{array}{cc} Y_{u}:\;\text{increases}\;\text{by}\;\text{1} & \text{at}\;\text{rate}\;\alpha \left(u\right)\sum_{v\in N_{in}\left(u\right)}Y_{v} \end{array}$$ (3)

$$\begin{array}{cc} Y_{u}:\;\text{decreases}\;\text{by}\;\text{1} & \text{at}\;\text{rate}\;\delta Y_{u} \end{array}$$ (4)

**Lemma 1** The process **Y(t)** stochastically dominates **X(t)** so that *X*(*t*) ≤ *Y*(*t*) *for all t* ≥ 0.

**Proof 1** Our proof is based on designing a coupling that ensures that *X*(*t*) ≤ *Y*(*t*) for all *t* ≥ 0, and builds on [17]. Clearly, *X*(0) ≤ *Y*(0). We consider the process **Y(t)** and for each node *u*, we sample random variables $R_{u}^{1}$ and $R_{u}^{2}$ from exponential distributions with parameters *α*(*u*)∑_*v* ∈ *N*_*in*_(*u*)_ *Y*_*v*_ and *δY*_*u*_, respectively. The first transition out of *Y*(0) happens at time *τ*, which equals ${\text{min}}_{u}\{R_{u}^{1},R_{u}^{2}\}$. Our coupling will specify the transition for the process **X(t)** in the following manner. Suppose the transition at time *τ* corresponds to *Y*_*u*_(*τ*) = *Y*_*u*_(0) + 1; this would have happened with rate *α*(*u*)∑_*v* ∈ *N*_*in*_(*u*)_ *Y*_*v*_(0). For the corresponding process **X(t)**, the transition *X*_*u*_(*τ*) = *X*_*u*_(0)+1 is made with probability $\frac{1}{\sum_{v\in N_{in}(u)}Y_{v}(0)}$, if {*v* ∈ *N*_*in*_(*u*):*X*_*v*_ > 0} ≠ *ϕ*; otherwise *X*_*u*_(*τ*) = *X*_*u*_(0). This ensures that the transition *X*_*u*_(*τ*) = *X*_*u*_(0)+1 happens with the correct rate. Similarly, the transition corresponding to *Y*_*u*_(*τ*) = *Y*_*u*_(0)−1 can be handled to get a coupling of the first jumps in **X(t)** and **Y(t)**.

**Lemma 2** Let *ρ*(*A*) denote the spectral radius of *A*, the adjacency matrix of *G*. Assume that *G* is a bi-directed graph and let *α*_*max*_ = max_*u*_ *α*(*u*). If *α*_*max*_ *ρ*(*A*) < *δ*, the duration of the cascade *T* satisfies Pr[*T* > *t*] ≤ *ne* ^−(*δ*−*α*_*max*_*ρ*(*A*))*t*^ and $\text{E}[T]\leq \frac{\text{log}n+1}{\delta -\alpha_{max}\rho (A)}$.

**Proof 2** Our proof is an adaptation of that of [17] for the SIS model; we describe it here completely for completeness. From equations (3, eqn:y2), it follows that

$$\begin{array}{ccc} E[Y_{u}\left(t+dt\right)-Y_{u}\left(t\right)|Y\left(t\right)] & = & \alpha \left(u\right)\sum_{v\in N_{in}\left(u\right)}Y_{v}\left(t\right)dt-\delta Y_{u}\left(t\right)dt+o\left(dt\right)\\ & \leq & \alpha_{max}\sum_{v\in N_{in}\left(u\right)}Y_{v}\left(t\right)dt-\delta Y_{u}\left(t\right)dt+o\left(dt\right), \end{array}$$

which implies $\frac{dE[\text{Y}(t)]}{dt}\leq (\alpha_{max}A-\delta I)E[\text{Y}(t)]$.

This has solution *E*[**Y**(*t*)] ≤ *e* ^(*α*_*max*_*A*−*δI*)^ **Y**(0). From Lemma 1, and since **X**(0) = **Y**(0), we have *E*[**X**(*t*)] ≤ *e* ^(*α*_*max*_*A*−*δI*)^ **X**(0).

Let *N*_*t*_ = ∑_*v*_ *X*_*v*_(*t*) = **1** ^*T*^ **X**(*t*) denote the number of nodes infected at time *t*. Then, *N*_*t*_ ≤ **1** ^*T*^ *e* ^(*α*_*max*_*A*−*δI*)^ **X**(0). Since *A* is a symmetric matrix, *e* ^(*α*_*max*_*A*−*δI*)^ is also symmetric, and we have $∥e^{\left(\alpha_{max}A-\delta I\right)}\text{X}(0)∥\leq \rho (e^{\left(\alpha_{max}A-\delta I\right)})∥\text{X}(0)∥=e^{\alpha_{max}\rho (A)-\delta }\sqrt{n}$. This implies *E*[*N*_*t*_] ≤ *ne* ^*α*_*max*_*ρ*(*A*)−*δ*^ = *ne* ^−(*δ*−*α*_*max*_*ρ*(*A*))^, since $∥\text{X}(0)∥\leq \sqrt{n}$. The first part of the lemma follows since Pr[*T* > *t*] = Pr[*N*_*t*_ ≥ 1] ≤ *E*[*N*_*t*_].

For the second part of the lemma, we have

$$\begin{array}{ccc} E[T] & = & \int_{0}^{\infty }\mathrm{Pr}\left[T>t\right]dt\\ & \leq & \int_{0}^{\infty }\min\left\{1,ne^{\alpha_{max}\rho \left(A\right)-\delta }\right\}\\ & \leq & \int_{0}^{\log n/(\delta -\alpha \rho (A))}1dt+\int_{\log n/(\delta -\alpha_{max}\rho \left(A\right))}^{\infty }ne^{-(\delta -\alpha_{max}\rho \left(A\right))}dt\\ & \leq & \frac{\log n+1}{\delta -\alpha_{max}\rho \left(A\right)} \end{array}$$

Lemma 2 implies that when *α*_*max*_ *ρ*(*A*) < *δ*, any cascade has size *O*(log*n*). We are able to prove Lemma 2 only when *G* is symmetric, because the proof relies on all eigenvalues being real, though the statement might be true in general.

#### 3.2.3 Conditions for Large Cascades

We now consider the conditions for having a large cascade (of size *c* ^*m*^, where *c* is a constant larger than 1, and *m* is a parameter). We need the following version of the isoperimetric constant, which captures node expansion.

$$\begin{array}{c} \hat{\eta }\left(G,m\right)=\min_{S\subseteq V,|S|\leq m}\frac{\sum_{v\in V-S:N_{in}\left(v\right)\cap S\neq \phi }\alpha_{v}}{\left|S\right|}. \end{array}$$

We sometimes omit the reference to the graph *G* in $\hat{\eta }(G,m)$, and just use $\hat{\eta }(m)$ when *G* is clear from the context. We now consider a Markov process **Z**(*t*) with state space {0, …, *m*}, defined in the following manner:

$$\begin{array}{ccc} Z(t) & = & Z\left(t\right)+1\;\text{at}\;\text{rate}\;\hat{\eta }\left(m\right)Z,\;\text{if}\;Z<m\\ Z(t) & = & Z(t)-1\;\text{at}\;\text{rate}\;\delta Z,\;\text{if}\;Z>0 \end{array}$$

**Lemma 3** *Z*(*t*) is stochastically dominated by ∑_*u*_ *X*_*u*_(*t*), i.e., *Z*(*t*) ≤ ∑_*u*_ *X*_*u*_(*t*) for all *t* ≥ 0.

**Proof 3** The proof is also by designing a coupling, as in Lemma 1. We assume that *Z*(0) ≤ ∑_*u*_ *X*_*u*_(0), and prove the statement by induction. We consider the process **X**(*t*) and for each node *u*, we sample random variables $R_{u}^{1}$ and $R_{u}^{2}$ from exponential distributions with parameters *α*(*u*)1_{*v* ∈ *N*_*in*_(*u*):*X*_*v*_ > 0} ≠ *ϕ*_ and *δX*_*u*_(0), respectively. The first transition out of *X*(0) happens at time *τ*, which equals ${\text{min}}_{u}\{R_{u}^{1},R_{u}^{2}\}$. Our coupling will specify the transition for the process **Z(t)** in the following manner. Let *S* = {*w*:*X*_*w*_(0) > 0}. Let *N* ^+^(*S*) = {*v* ∈ *V*−*S*:*N*(*v*)∩*S* ≠ *ϕ*}.

Suppose the transition at time *τ* corresponds to a transition *X*_*u*_(*τ*) = *X*_*u*_(0)+1 for some node *u* (which increases the number of active messages). The total rate at which such an increase happens equals ∑_*u*_ *α*(*u*)1_{*v* ∈ *N*_*in*_(*u*):*X*_*v*_ > 0} ≠ *ϕ*_ = ∑_*u* ∈ *N*^+^(*S*)_ *α*(*u*). First, suppose that ∣*S*∣ < *m*. The transition *Z* = *Z*+1 is now made at time *τ* with probability

$$\begin{array}{c} \frac{\hat{\eta }\left(m\right)Z}{\sum_{u\in N^{+}\left(S\right)}\alpha \left(u\right)}. \end{array}$$

This fraction is in [0, 1], because $Z(0)\hat{\eta }(m)\leq \sum_{u\in N^{+}(S)}\alpha (u)$, by definition of $\hat{\eta }(m)$, and because ∣*S*∣ < *m*, so that the transition happens with the correct rate. Second, if ∣*S*∣ ≥ *m*, *Z* is unchanged, which is the correct rate.

Next, we consider the case that the transition at time *τ* corresponds to a transition *X*_*u*_(*τ*) = *X*_*u*_(0)−1 = 0 for some node *u*. In this case, the transition *Z*(*τ*) = *Z*(0)−1 is made with probability $\frac{Z(0)}{\sum_{u}X_{u}(0)}$, which is well defined since this is in [0, 1]. Also, note that there is some probability that ∑_*u*_ *X*_*u*_(0) decreases by 1, but *Z*(0) does not— this does not violate the property, because in this case *Z*(0) < ∑_*u*_ *X*_*u*_(0).

Therefore, in either case, we have *Z*(*τ*) ≤ ∑_*u*_ *X*_*u*_(*τ*), and the lemma follows.

**Lemma 4** Suppose $r=\frac{\delta }{\hat{\eta }(m)}<1$. Then, we have $\text{Pr}[T>\frac{r^{-m+1}}{2m}]\geq \frac{1-r}{e}(1+O(r^{m}))$.

**Proof 4** The proof of the above lemma follows by observing that the process **Z**(*t*) is a one-dimensional random walk, defined in the following manner. Consider the discrete time Markov chain associated with *Z*. Let *p*(*i*, *j*) denote the probability that *Z* switches to value *j* from *i*. Then, we have:

$$\begin{array}{ccc} p(i,i+1) & = & \frac{\hat{\eta }\left(m\right)}{\hat{\eta }\left(m\right)+\delta },\;i=1,\cdots ,m-1,\\ p(i,i-1) & = & \frac{\delta }{\hat{\eta }\left(m\right)+\delta },\;i=1,\cdots ,m-1\\ p(0,0) & = & 1,\\ p(m,m-1) & = & 1. \end{array}$$

Then, the duration of the cascade, *T*, is the time before the process hits 0. As in [17], this is the standard gambler’s ruin probability, and the rest of the proof follows exactly as in [17].

**Spectral connection**. Vertex expansion is related to the graph spectrum. If *G* is a *d*-regular graph, and if its spectral gap, i.e., the difference between the smallest and second smallest eigenvalue, is *μ*, the vertex expansion for sets of size at most *m* is $\frac{1}{(1-m/n)\mu^{2}+m/n}$.

#### 3.2.4 Identifying Critical Sets in a Cascade

We now consider the following questions: What is the critical subset of users whose tweets are responsible for the cascade to survive? What is the critical subset of users whose removal would cause the cascade to disintegrate? These are related and complementary problems, which can help explain the conditions for cascade formation. We consider a slightly more general notion of cascades than the one defined in Section 3.1—for a set *S* of nodes, we define *C*(*S*, *t*, Δ) = ∪_*u* ∈ *S*_ *C*(*u*, *t*, Δ) to be the union of cascades starting at nodes in *S*. As a result, any directed acyclic graph can be seen as a cascade formed by its sources.

CriticalSetShattering Problem CSSP(*G*, 𝓒, *k*):

*Input*: A set of cascades 𝓒 in a graph *G* = (*V*, *E*) and parameter *k*.

*Goal*: Determine the smallest set *S* ⊆ *V* of users, such that the sub-cascades of all *C* ∈ 𝓒 in *G*[*V*\*S*] are of size at most *k*.

Thus, the goal in CSSP is to find the subset *S* whose removal causes all cascades in 𝓒 to be “shattered”.

CriticalSetFormation Problem, CSFP(*G*, 𝓒, **α**, *k*):

*Input*: A set of cascades 𝓒 in a graph *G* = (*V*, *E*), tweet rate **α** and parameter *k*.

*Goal*: Determine the smallest set *S* ⊆ *V* of users, such that for every *C* ∈ 𝓒, a sub-cascade of size at least *k* exists in the graph *G*[*S*] with tweet rate **α**.

Thus, CSFP quantifies the number of users needed to cause large cascades. While CSSP and CSFP are closely related and seem to be complementary problems, they are quite different from a computational perspective.

**Complexity and algorithms for** **CSSP**. We have the following result.

**Lemma 5** CSSP (*C*, *G*, *k*) is NP-complete.

**Proof 5** It is easy to verify that CSSP is in NP. The NP-hardness of CSSP is by a reduction from the balanced graph partitioning problem (see, e.g., [37])— this problem involves finding the smallest subset *S* ⊂ *V*′ of nodes in an undirected graph *H* = (*V*′, *E*′) so that all components in *H*[*V*′−*S*] have size at most *b*, which is a given parameter.

Let *C* be a DAG formed by orienting the edges of *H* arbitrarily, so that it forms a DAG. Let *B* ⊂ *V*′ whose removal splits *C* into weakly-connected components *H*_1_, …, *H*_*r*_, each of size at most *k* = *b*; as discussed earlier, each component *H*_*i*_ is a cascade formed by the sources in that DAG. If we ignore the directions of the edges, we get components of size at most *k* = *b*. This implies that the solution to CSSP in *C* corresponds to a solution to the separator problem on *H*. The converse also holds similarly.

Whenever the condition in Lemma 2 is tight, i.e., it gives both necessary and sufficient conditions, CSSP can be solved by simply attempting to reduce the spectral radius *ρ*(*A*). We consider the special case of the Chung-Lu random graph model [38]: given a weight sequence **w** = (*w*(*v*_1_), *w*(*v*_2_), ⋯, *w*(*v*_*n*_)) for nodes *v*_*i*_ ∈ *V*, the random graph *G* ∈ *G*(**w**) is obtained by choosing each edge (*u*, *v*) with probability $\frac{w(u)w(v)}{\sum_{v_{i}\in V}w(v_{i})}$. We use the following result from [39].

**Lemma 6** *[39] If *G* = *G*(**w**) is a random graph in the Chung-Lu model with the weight sequence being a power law with exponent β* > 2, *removal of the* Θ(*n*/*T* ^2(*β*−1)^) *nodes with the highest weight ensures that the spectral radius of the residual graph is at most T, almost surely*.

Motivated by Lemma 6, we study heuristics for CSSP based on degree and the core number in the underlying graph. Since $\rho (A)\geq \sqrt{{\text{max}}_{v}deg(v,G)}$, a natural heuristic for CSSP is to reduce the maximum degree max_*v*_ *deg*(*v*, *G*). Also, since $\rho (A)\geq \frac{2|E(H)|}{|V(H)|}$ for any subgraph *H* of *G*, another natural heuristic for CSSP is to reduce the density of every subgraph *H*. Motivated by these bounds on the spectral radius of a graph, we consider the following heuristics for CSSP: (*i*) *high degree heuristic*: remove nodes in decreasing order of degree in *G*; and (*ii*) *high core number heuristic*: remove nodes in decreasing order of their core-number in *G*.

**Complexity and algorithms for** **CSFP**. We have the following hardness result.

**Lemma 7** CSFP (*C*, *G*, **α**, *k*) is NP-complete.

**Proof 7** It is easy to verify that CSFP is in NP. We only discuss the NP-hardness. Our proof is by a reduction from the Set Cover problem, an instance of which consists of a set *B* of elements, a set *A* of subsets of *B*; the goal is to select the smallest subset *A*′ ⊂ *A* such that each element in *B* is covered by a set in *A*′.

We construct an instance of CFP in the following manner. We set *ϵ* to be a large integer. We construct a graph *G* = ({*r*, *r*′}∪*A*∪*B*, *E*), where *E* consists of the following edges: edges (*j*, *i*) if *j* ∈ *B*, *i* ∈ *A* and *j* is contained in set *i*, edges (*r*, *i*), (*r*′, *i*) for all *i* ∈ *A*, and edge (*r*, *r*′). We have *α*_*r*_ = *α*_*r*′_ = *ϵ*, while *α*_*u*_ = 1/*n* for all *u* ∈ *A*∪*B*. We note that $\eta (G,\hat{1},\{u\})\geq \epsilon$ for all *u* ∈ {*r*, *r*′}∪*A*.

Suppose *A*′ ⊆ *A* is a minimum set cover. Then, increasing *α*_*u*_ = *ϵ* for all *u* ∈ *A*′ will ensure that $\eta (G,\hat{1},\{j\})\geq \epsilon$ for all *j* ∈ *B*. Similarly, suppose *S* is the optimum solution to the CFP problem. Clearly, *S* ⊆ *A*; if *S*∩{*r*, *r*′} ≠ *ϕ*, we can drop *r*, *r*′ from *S* without affecting the feasibility of the solution. For each *j* ∈ *B*, there must be at least one neighbor in *S*; else, we cannot have $\eta (G,\hat{1},\{j\})\geq \epsilon$. This implies *S* is a set cover.

This completes the reduction.

We consider a greedy algorithm for CSFP: pick nodes in non-increasing order of degree until the cascade on the graph induced by these nodes has size at least *k*.

We note that the maximum cascade size can be estimated for a given rate assignment within a factor of 1±*ϵ*, with high probability, in time *O*(∣*E*∣log*n*/*ϵ* ^2^) by a standard Chebyshev bound.

### 3.3 Forecasting Social Unrest using Cascades

Social media is believed to be responsible for facilitating critical communication often required to fuel momentum preceding the events of civil unrest. Here we explore the ability of Twitter data to act as a predictive signal of future civil unrest. Specifically, we study the prediction of civil unrest events (e.g., protests, strikes) by using properties of the activity cascades in Twitter data.

We employ a regression model to predict the probability of a civil unrest event in a given day by using features based on the structural properties of the activity cascades described earlier. We hypothesize that, in general, unusually large and long cascades are likely to be indicative of future events of interest. These could be sport events, concerts, revolutions, elections, etc. However, *given that our tweets are filtered by civil unrest related keywords, we expect the events detected will be of civil unrest type.*

Starting from May 1, 2012 to November 30, 2013, each day, we compute the total number and size of cascades, number of participants and duration (in days) of cascades, change in the number of participants and tweets, average growth rate of tweets and average growth rate of participants. These features are collected daily for each active follower cascade and MRT cascade. For each of the features described above, we also compute the minimum, maximum, median, and average of the cascade size, duration, and users, as well as the average value of the 1st, 2nd, 3rd, and 4th quartile of their distribution and add them to the feature set. Therefore, our initial feature set consists of 114 attributes: (7 cascade properties × 8 aggregate statistics × 2 types of cascades + (daily cascade count × 2 types of cascades)).

Our initial list of features is expected to be highly correlated, resulting in unstable estimates if used in regression modeling. For example, the cascade size of an MRT cascade is almost equivalent to the number of users in that cascade, since people tend to retweet a message only once. In addition, the majority of these cascades last for one day (duration = 1), which makes the average growth equivalent to the change in the cascade size on the last day, which in turn gives the change in the number of users, and so on.

In order to address the problem of multi-colinearity, we compute the correlation between every pair of features (i.e. the correlation matrix) and remove highly correlated features. Specifically, if the correlation between two variables is greater than 0.7, we only keep one of the two. This methodology reduced the size of our feature set from 114 to 13. Next, we use a regression methodology called LASSO (Least Absolute Shrinkage and Selection Operator) to further remove the redundant features and to shrink coefficients [40]. The LASSO based logistic regression model uses cascade features from both the follower and the MRT models.

#### 3.3.1 Baseline model

We build a baseline model in order to set a benchmark and to measure the added predictability provided by the Twitter data. The baseline model uses no external input in the regression model. It uses an autoregressive logistic model of order 1, since we have a binary dependent variable. It uses lagged values of itself as the predictor. Formally, we estimate *Y*_*t*_ = *α* + *βY*_*t*−1_ + *ɛ* where *Y*_*t*_ is the binary variable: 1, if there is an event on day *t* in the GSR; 0 otherwise. The fitted values of *Y*_*t*_, which give the likelihood of future events, are compared against the actual events in the GSR for measuring the model’s performance.

#### 3.3.2 Evaluation metrics

Once the probabilities are estimated for the test days, a threshold *t* is used to determine whether or not the probability exceeds *t* for an event to occur. The optimal threshold t* is determined by cross-validation, especially maximizing the area under the ROC (receiver operating characteristic) curve. Once learned, t* is further used to separate events from non-events given the estimated probabilities. We evaluate our models against two settings—a lead time of 1 day and a lead time of 2 days—and compare the results against the GSR using standard measures such as precision, recall, and the misclassification rate.

#### 3.3.3 Operational issues

All the Twitter data used in this study is in compliance with the Terms of Use and all website conditions of Twitter. We use data from May 2012 to infer the activity cascades. The GSR data is available only from Nov 2012. Hence, data from Nov 2012 to May 2013 is used for training the forecasting model and the held-out June 2013 data is used for testing in case of Brazilian Spring. This training/test split is actually a tough test for the forecasting algorithm because June 2013 depicted very significant changes in rates of occurrences of protests in Brazil (more on this below), and our approach was nevertheless able to forecast this variation in its forecasts.

## 4 Results and Discussion

Our experimental results are focused on answering the following questions.

1. Do our theoretical conditions for large cascades from Section 3.2 hold true in real datasets? (Section 4.1)
2. What level of increase in user tweeting initiates large cascades? Does the solution to the CFP problem using our greedy heuristic suggest that a few important users are sufficient or is large number of users necessary? (Section 4.2)
3. How adept are activity cascades at detecting precursors and surrogates for protests? (Section 4.3)
4. Which cascade features yield the best forecasting performance? Are these features consistently better across multiple countries? (Section 4.4)
5. Are cascade models for forecasting protests significantly better than baseline models? Is there value in building features from different Twitter networks (i.e. follower and MRT)? (Section 4.5)
6. Can our methods help forecast ‘black swan’ events like the Brazilian Spring? (Section 4.6)

### 4.1 Validation: Two Regimes for Cascade Sizes

**Fig 2:**
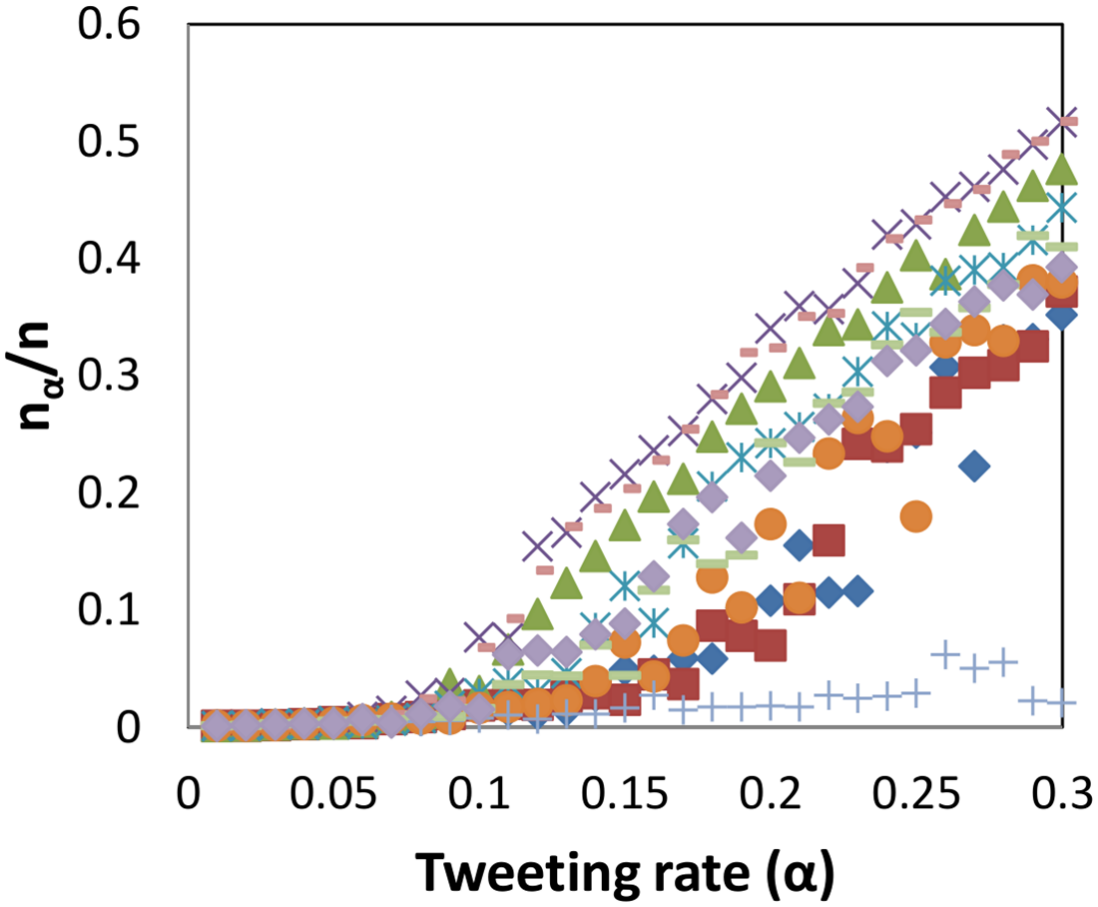
Follower cascade size as a function of tweeting rate for ten follower cascades in Mexico (produced between June 27, 2012 and September 7, 2012), with synthetic traffic. As the tweet rate of the users increases, we observe a sudden transition from a regime of very low user participation to a higher-activity regime.

We empirically verify the conditions for large cascades uncovered using our theoretical analysis. We find ten of the largest follower cascades in Mexico between June 27 and Sep 7, 2012, and the subgraphs induced by these users (also referred to as the cascade graphs). We consider synthetic twitter traffic generated using a Poisson process (as in Section 3.2) for the users in these cascade graphs with rate *α*_*u*_ = *α*, and then compute the cascades induced by this for Δ = 4 hours. Fig 2 shows the maximum cascade size *n*_*α*_, as a function of *α* for each of these cascade graphs. The y-axis is normalized by the number of nodes *n* in the respective cascade. For most of the cascades, we observe a clear phase transition for *α* somewhere in the range [0.05,0.15]. For these particular graphs, we find that $\rho (\hat{A})$ (in the notation of Lemma 2) is below *δ* when *α* is in the range [0.10,0.15]. We also observe in Fig 2 that the cascades die out when *α* ≤ 0.05, which is consistent with the condition in Lemma 2. Note that some of the cascades die out even for higher values of *α*, which is consistent with the gap between the necessary and sufficient conditions in Lemmas 2 and 4.

### 4.2 Identifying Critical Sets in Cascades: CSSP and CSFP

**Empirical analysis of heuristics for** **CSSP**. We start with collections of tweets from Brazil, Mexico, and Venezuela that form cascades, in monthly intervals, from May 2012 through July 2013. We use reciprocal follower graphs (i.e., two users must follow each other to form an edge in the reciprocal follower graph, which implies a stronger association between users [5]) to determine which users follow each other. We use Δ = 4 hours for the maximum duration that may separate a user’s and a follower’s tweets in forming edges in the cascade graph. The reciprocal follower graphs for Brazil, Mexico, and Venezuela have 1.9, 0.5, and 4.9 million edges, respectively, and 123409, 69226, and 253423 nodes.

**Fig 3:**
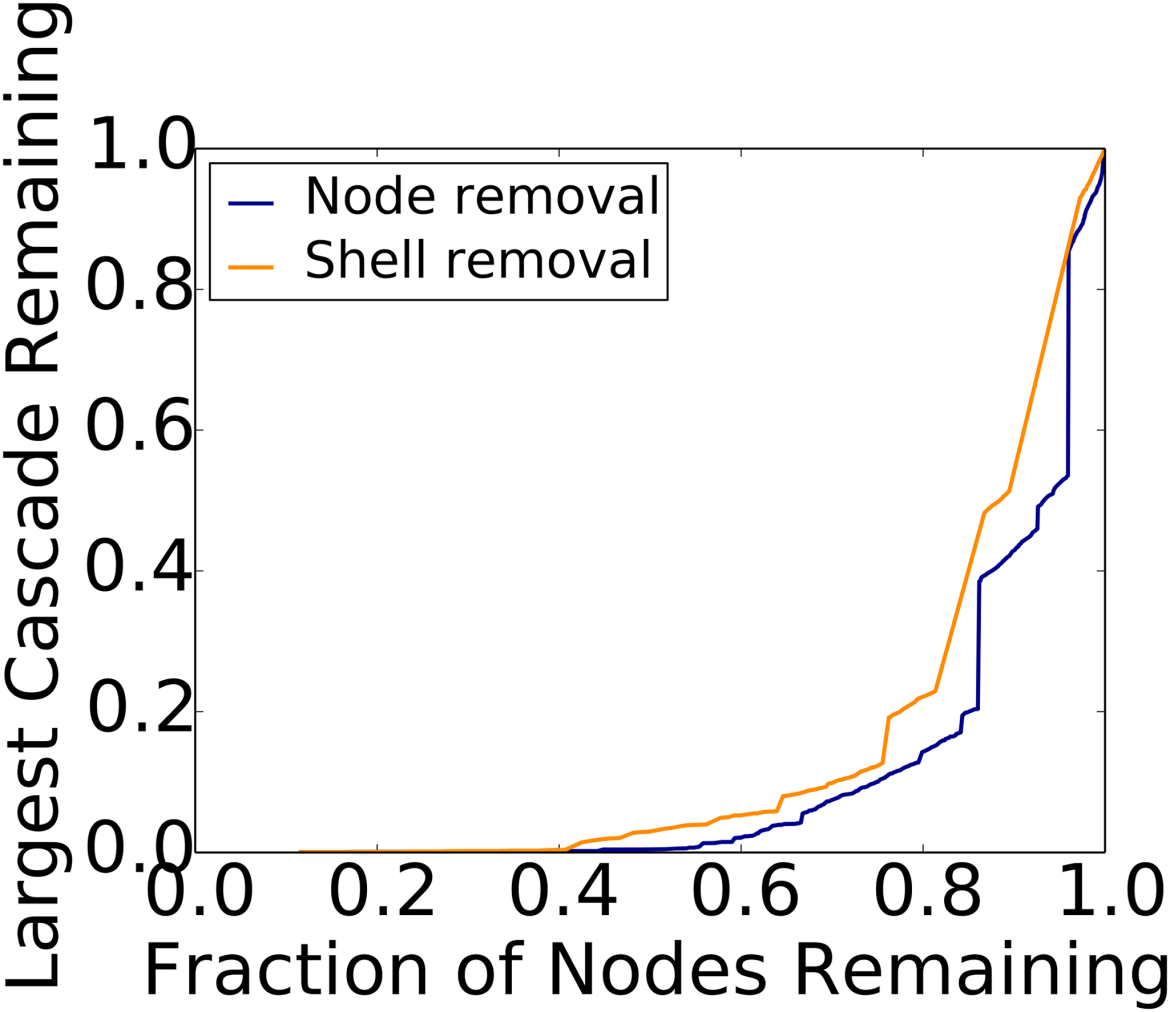
Node and shell removal heuristics for CSSP (Brazil). Here, we see the largest remaining sub-cascade size in terms of numbers of tweets (normalized by the original size) as a function of numbers of remaining nodes in the cascade graph (normalized by the original number of nodes). This cascade occurred in June 2013, and its original size is 15,791 tweets.

**Fig 4:**
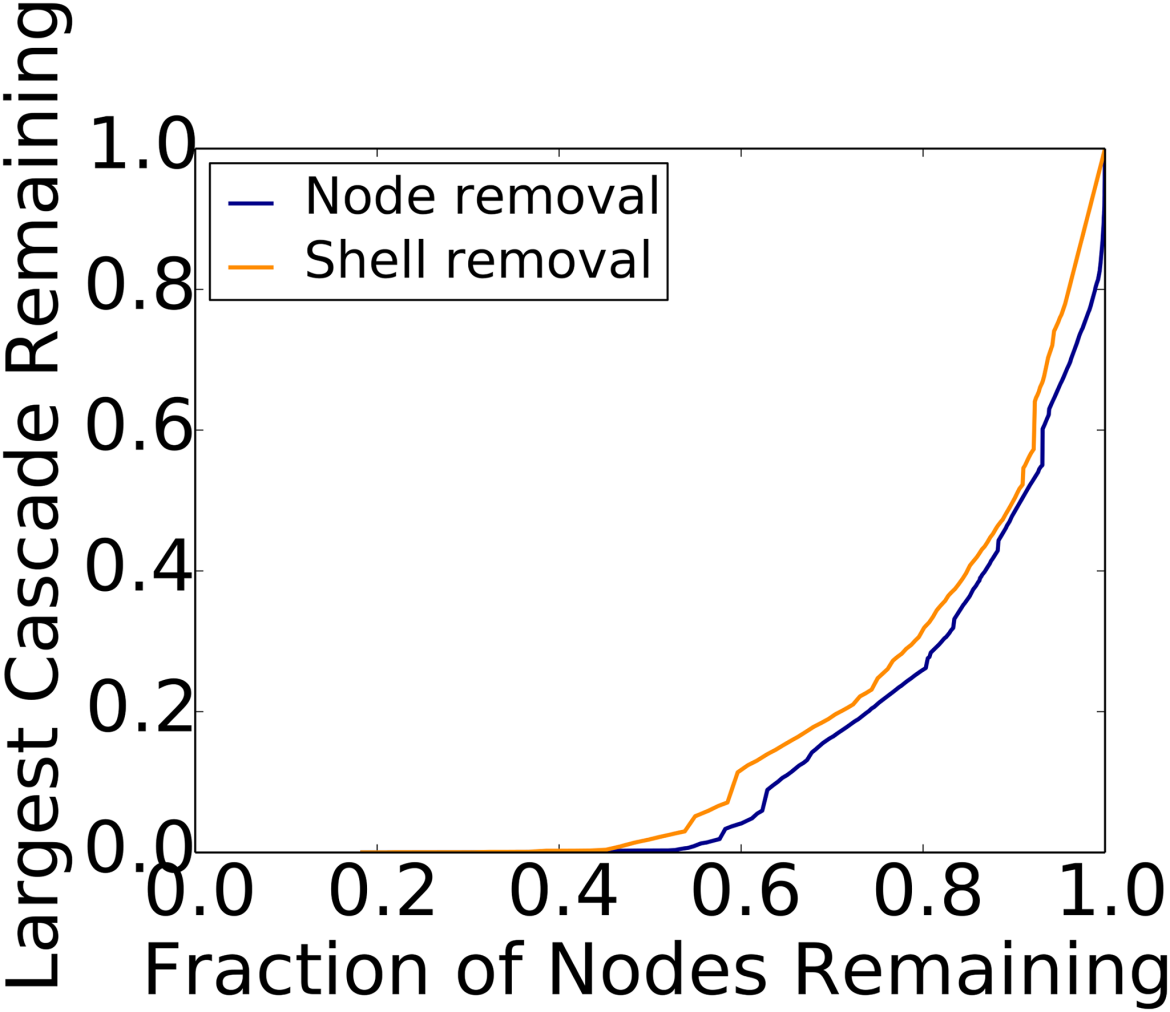
Node and shell removal heuristics for CSSP (Venezuela). Here, we see the largest remaining sub-cascade size in terms of numbers of tweets (normalized by the original size) as a function of numbers of remaining nodes in the cascade graph (normalized by the original number of nodes). This cascade occurred in April 2013, and its original size is 226,179 tweets.

We select nodes (users) from the cascade graphs based on node properties in the *follower graph*. Specifically, we successively remove nodes (*i*) from greatest degree to least, and (*ii*) from the greatest k-shell to the least, from the follower graphs. We then remove these nodes from cascade graphs and compute the numbers of nodes and the sizes of the largest weakly connected components that remain in them (cf. Section 3.2.4). Recall that nodes in a cascade graph are (user,time) pairs. Results are provided in Figs 3 and 4 for the largest cascades of Brazil and Venezuela, respectively. Results for other cascades, across countries and months, show the same behavior.

Removing relatively small fractions of high degree nodes and high k-shell nodes are both effective in reducing the sizes of cascades. For all (country, month) combinations, the high degree heuristic is more effective than the high k-shell heuristic. Differences between the methods can be significant, particularly for small numbers of removed nodes.

**Empirical analysis of heuristics for** **CSFP**. We now solve the CSFP problem for selected large cascades in Mexico, Brazil, and Venezuela using the greedy heuristic in Section 3.2.4, in order to approximate the change in the level of tweeting that caused the cascade. We examine the differences in aggregate level of tweets and user participation, as well the characteristics of cascades that might result at lower levels of participation. When we consider the largest cascades and retain either the tweets or the users involved with probability *p*, the resulting sub-cascade size varies quite gradually with *p*, instead of showing a clear phase transition (in contrast with the results in Section 4.1). It is possible that the more gradual change is due to the non-uniform rates *α*_*u*_ for users *u* in the large cascades, which cause a higher level of weighted vertex expansion, even for moderate values of *p*. These results are omitted because of space constraints.

**Fig 5:**
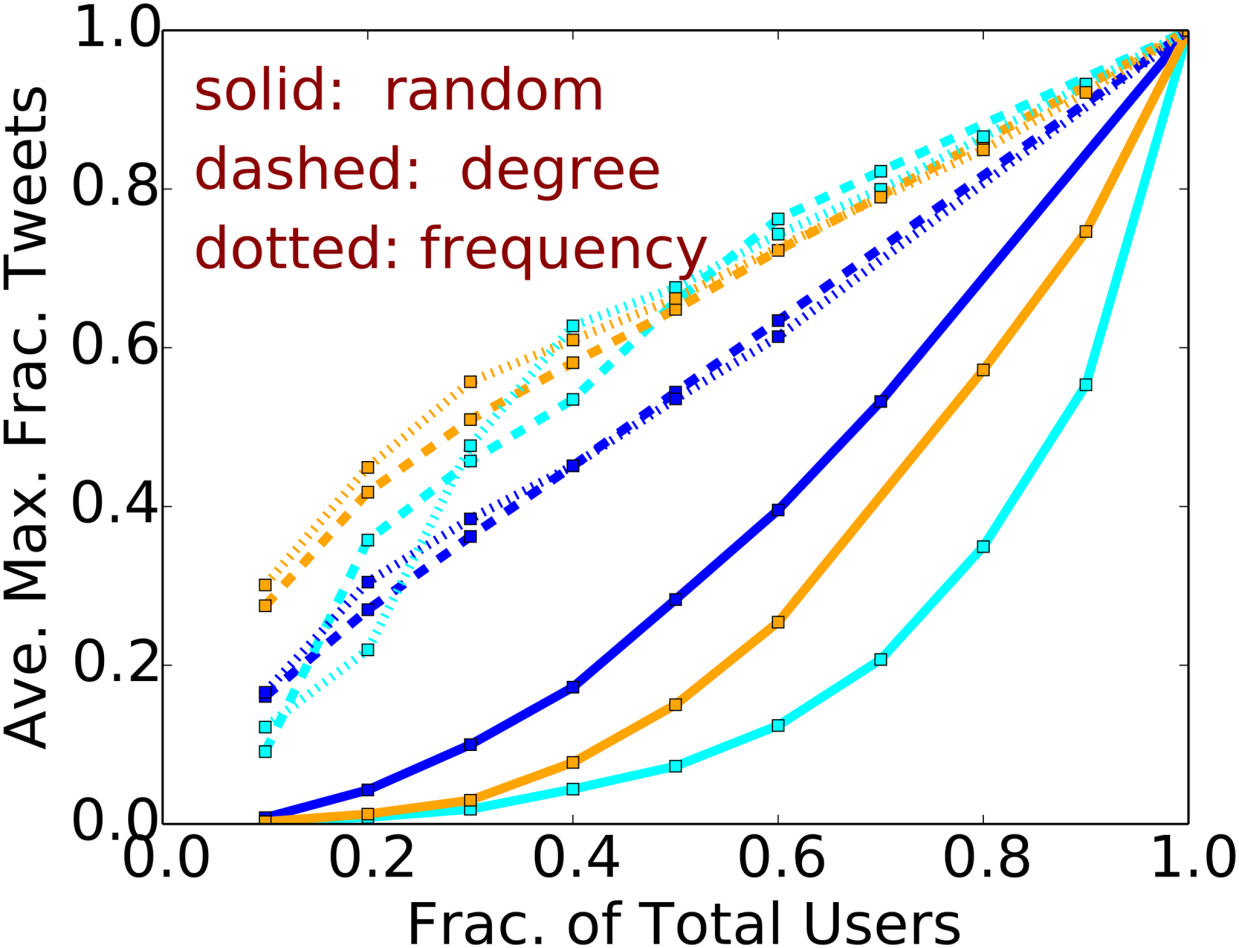
Greedy heuristic for CSFP. The (normalized) maximum cascade size vs. the fraction of users selected for some of the largest cascades in different countries. Data are: blue (Mexico, Δ = 1 hour); light blue (Brazil, Δ = 4 hours); and orange (Venezuela, Δ = 4 hours).

**Fig 6:**
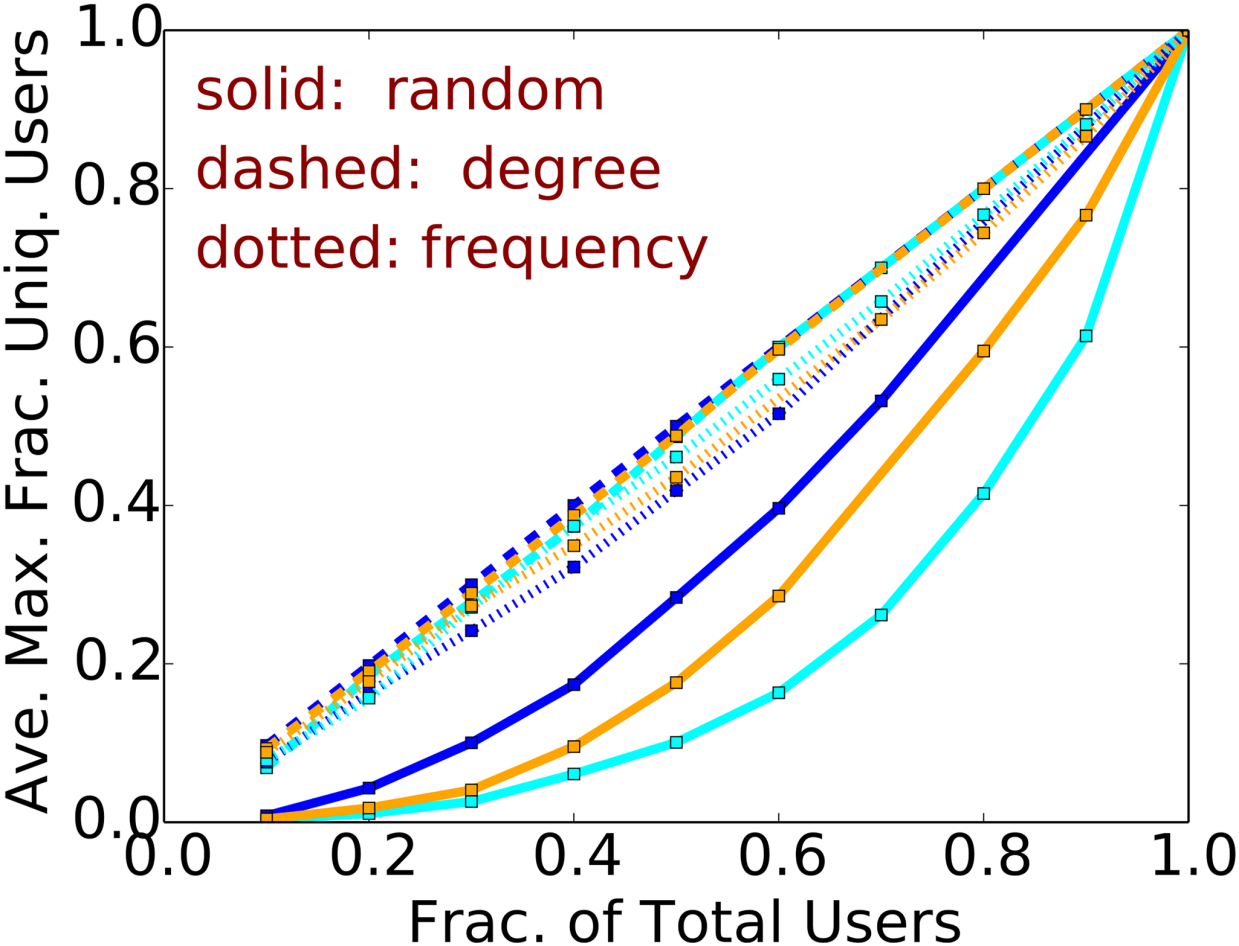
Greedy heuristic for CSFP. The (normalized) maximum number of unique users vs. the fraction of users selected for some of the largest cascades in different countries. Data are: blue (Mexico, Δ = 1 hour); light blue (Brazil, Δ = 4 hours); and orange (Venezuela, Δ = 4 hours).

We now consider the effect of a greedy choice of users from the original cascades, using variants of the greedy heuristic described for CSFP. The first (structural) heuristic selects *k* nodes {*v*_1_, …, *v*_*k*_} with the greatest values $|N_{o}^{\prime }(v)|$, where $|N_{o}^{\prime }(v)|$ is the number of out-neighbors of *v* appearing in the maximum cascade for a (country, Δ) pair; this is a high-degree heuristic. The second (dynamical) heuristic simply chooses the *k* nodes with the greatest frequency of occurrence in a cascade. In both heuristics, *k* = *pN*_*c*_, where *p* is the probability of selecting a node (cf. previous subsection) and *N*_*c*_ is the number of nodes in an original cascade, making it consistent with the earlier analysis. We compare these with a random selection of users with probability *p*. Fig 5 shows the (normalized) maximum size of a cascade for each of the above heuristics (labeled “degree”, “frequency” and “random”, respectively), averaged over 50 trials. Fig 6 shows the corresponding normalized maximum number of unique users in the cascades. The normalization constant in each plot is the empirically determined maximum cascade size and maximum number of users, respectively. We find that the high degree heuristic generally produces the largest cascades in terms of tweets and users. For ordinate values in the range 0.2 to 0.4, the maximum sizes for the high degree heuristic are 2× to 10× those of the random heuristic. These data indicate that large cascades are tenuous; e.g., even with the high degree heuristic, 80% of the original users are required to produce a cascade that is 80% of the maximum measured size. Thus, it is not the case that a few users drive cascade formation. However, for CSSP, removal of a smaller fraction (∼ 10–20%) of users can significantly reduce cascade size.

### 4.3 Illustrative Results of Tweet Contents

What types of tweets form large activity cascades that are predictive of protests? There are at least two broad classes of such tweets that we highlight here. The first kind pertains to tweets as an early reporting mechanism that then go on to form activity cascades that can serve as a protest recruitment or mobilization staging ground. The second kind are tweets that explicitly call for protest action by individuals.

As an example of the first kind, we discuss two tweets with a high retweet count found in our MRT cascades for Brazil. The original tweets were sent on January 27, and they are about a past event (night club fire) which led to the deaths of 231 people in Santa Maria. These tweets eventually formed part of a cascade that corresponded to an actual demonstration that took place on January 28, 2013. According to the news articles, 35,000 people marched and held a moment of silence in front of the gymnasium where the victims’ bodies had been identified. This shows that tweets selected by our vocabulary and tracked for activity cascade formation may indeed correspond to actual protest events on the ground. The second kind is highlighted by a tweet calling for a protest on September 7, illustrating that further analysis of tweets originating from such cascades can aid in forecasting.

### 4.4 Cascade Feature Utility for Forecasting

**Fig 7:**
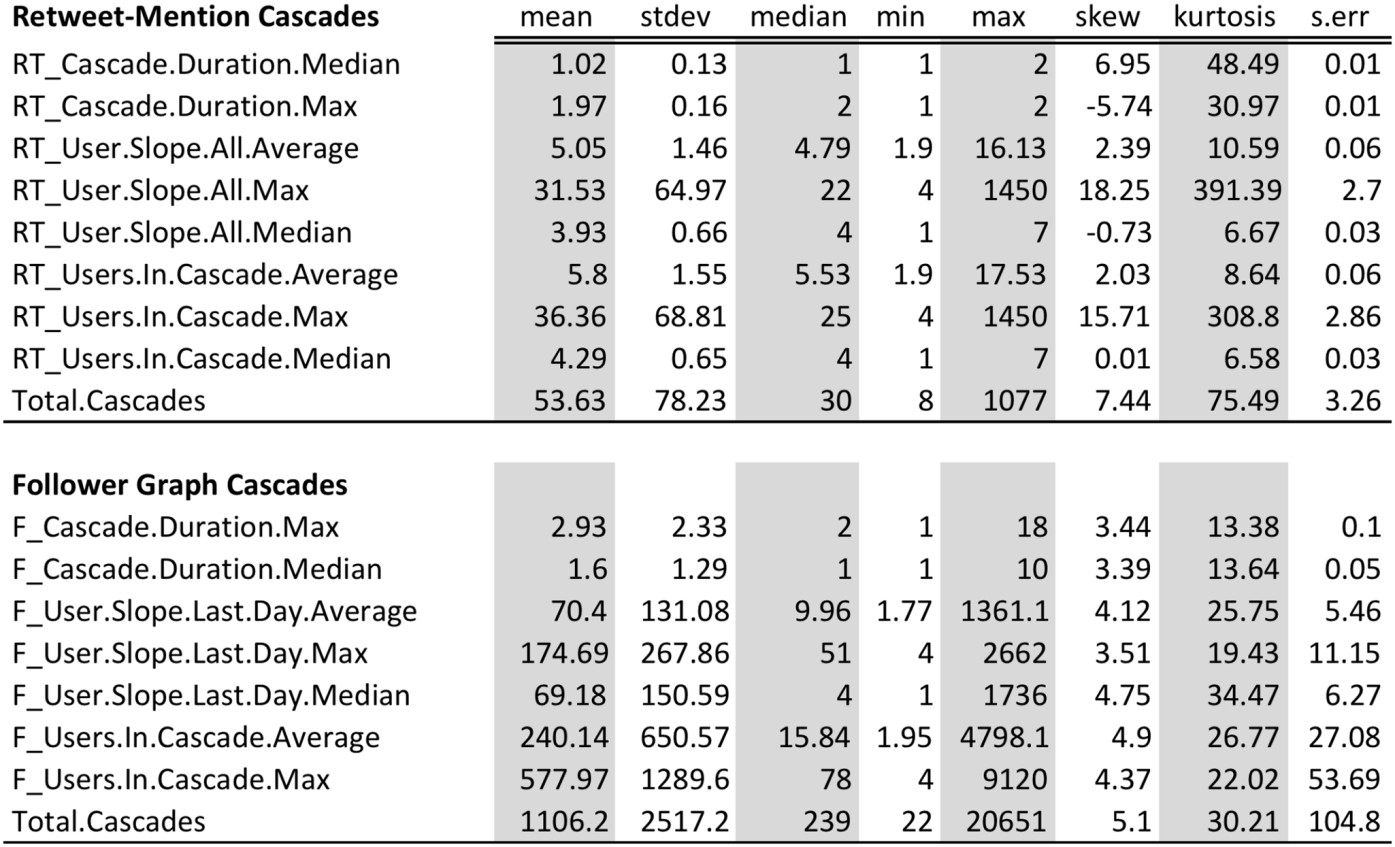
Descriptive statistics of selected features (Brazil) for the MRT and F models. The names in the first column consist of the name of the structural feature (i.e., cascade size, duration or slope, which is the incremental increase in the size per day), and the statistical operations (i.e. median, average etc.).

**Fig 8:**
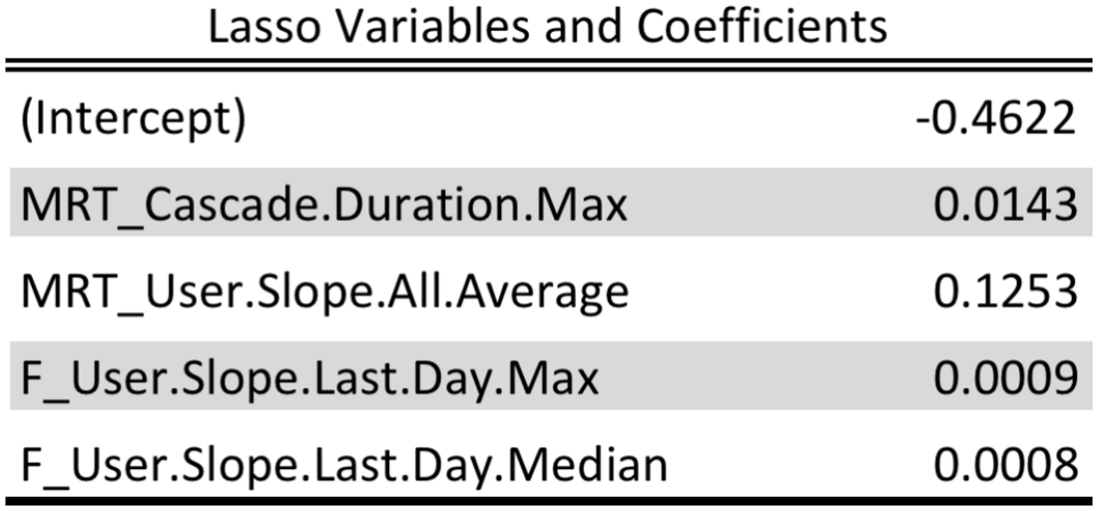
LASSO Variables. Variables selected by LASSO in the cascade model for Brazil, for a training period of November 2012 through May 2013.

Fig 7 illustrates descriptive statistics of selected features of mention and follower graph cascades in Brazil. Fig 8 shows the variables selected by the LASSO based logistic regression model. The LASSO based model finds that the probability of an event depends upon the duration and the slope of the follower and MRT graphs. These selected features are used as explanatory variables in a generalized linear regression model [41] which confirms their significance and relevance.

### 4.5 Comparison Against Baseline Models

**Fig 9:**
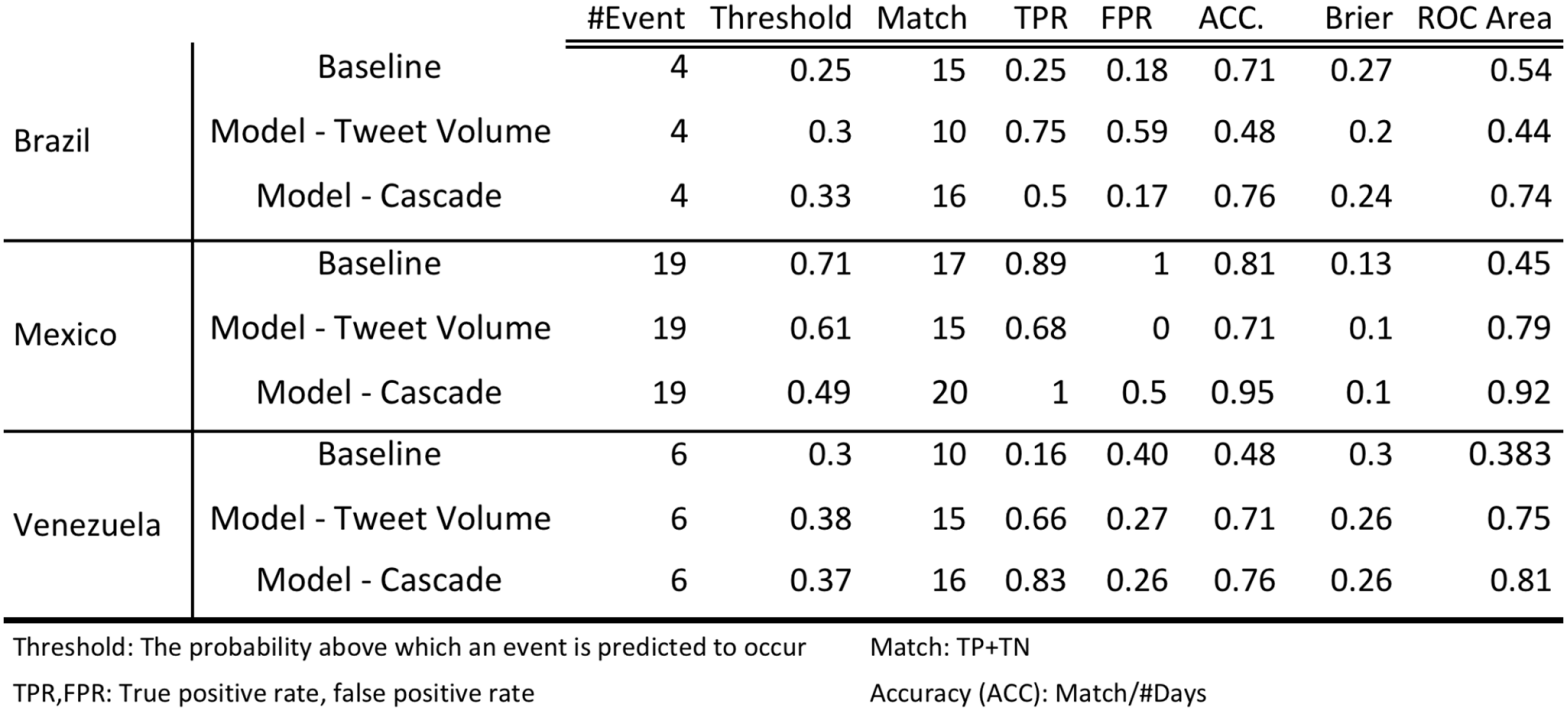
Performance of the predictive models. We show the performance of the three models in terms of accuracy, brier score, and area under the ROC curve. The cascades model has the best performance accross different countries.

Fig 9 compares the performance of the baseline model, volume-based model and the cascade model for the three countries. For each model, we report the threshold used, true positive rate (TPR), false positive rate (FPR), accuracy (ACC), brier score, and the area under the ROC curve. The results in Fig 9 report the threshold that results in the highest accuracy in prediction.

**Fig 10:**
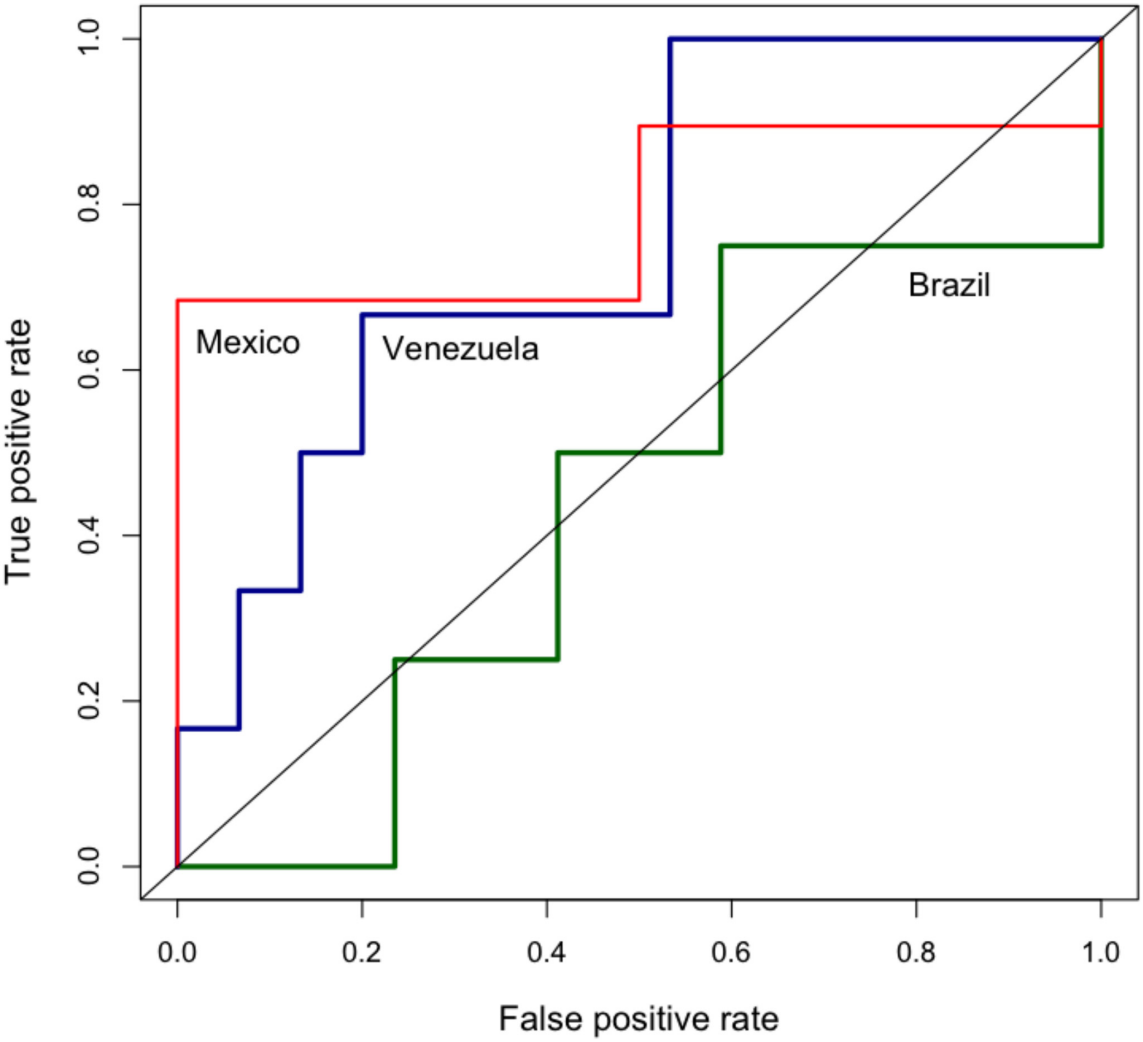
ROC curves for the volume-based model. We show the ROC curves for Mexico, Brazil, and Venezuela. Training period November 1, 2012 to November 9, 2013; test period November 10, 2013 to November 30, 2013.

**Fig 11:**
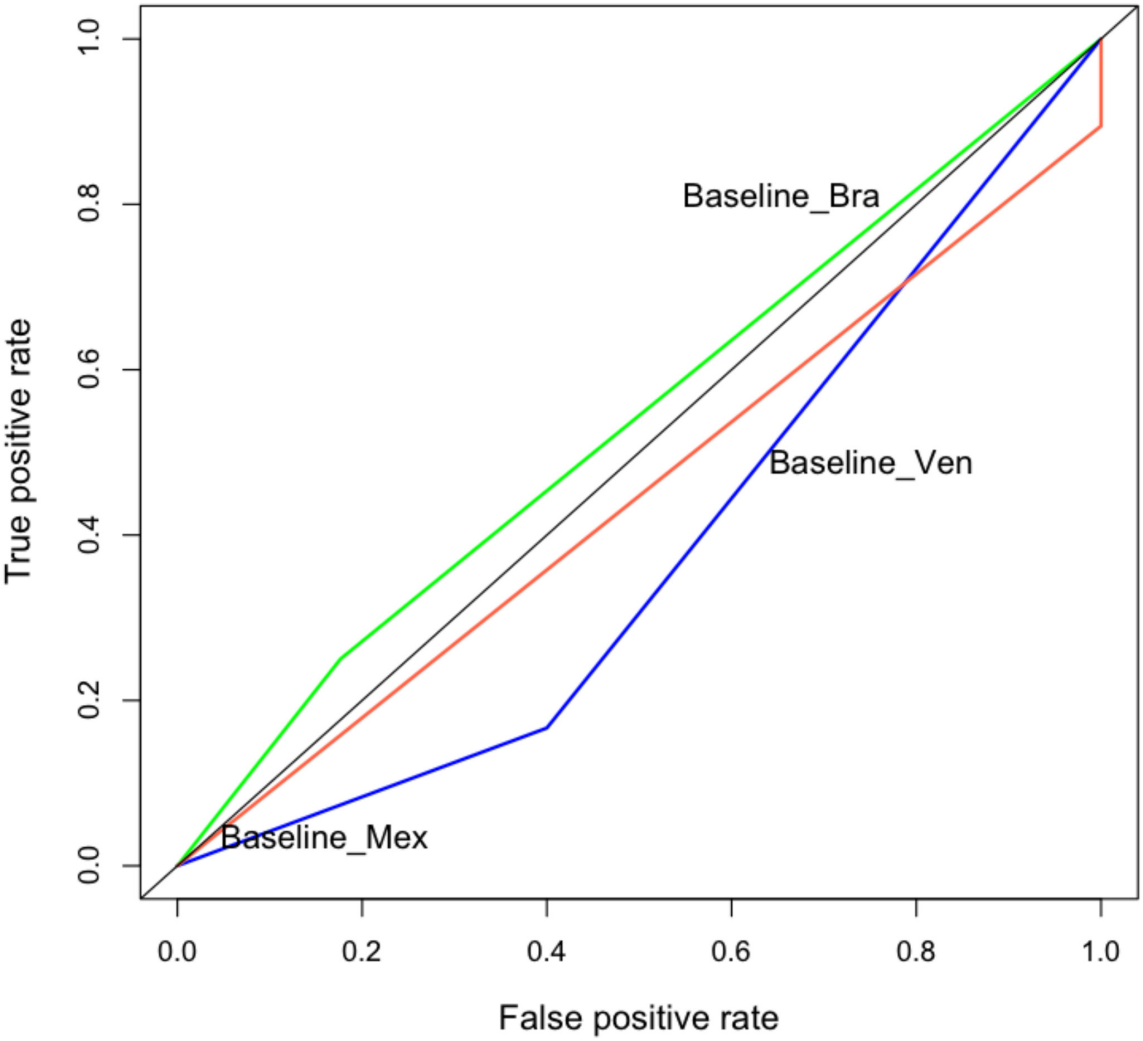
ROC curves for the baseline model. We show the ROC curves for Mexico, Brazil, and Venezuela. Training period November 1, 2012 to November 9, 2013; test period November 10, 2013 to November 30, 2013.

Note that the cascade model outperforms both the baseline model and the volume-based model. Figs 10 and 11 shows the ROC for these models. Each point in the line represents a different threshold for the model.

**Fig 12:**
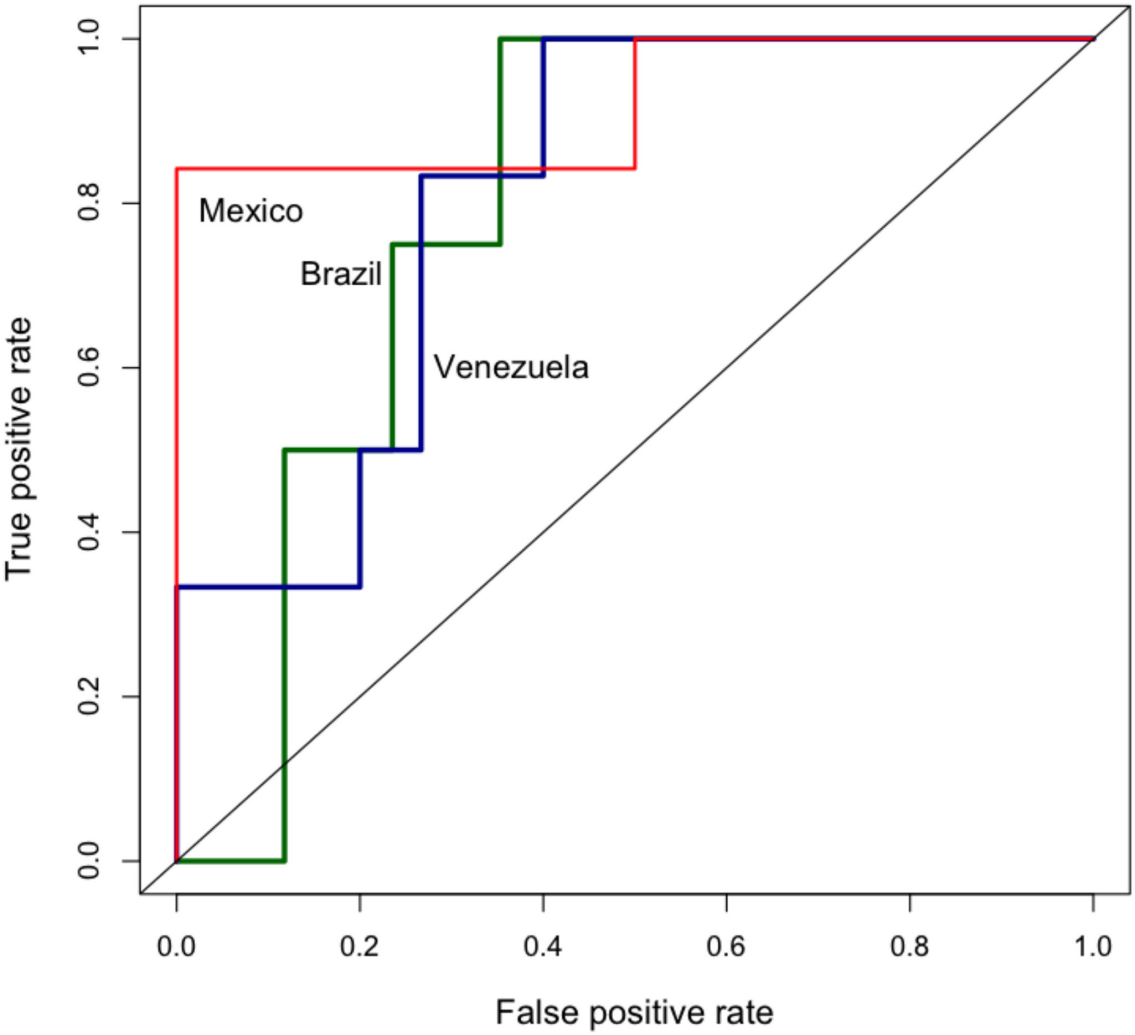
ROC curves for different countries. ROC curves for Mexico, Brazil and Venezuela for the cascade model. Training period November 1, 2012 to November 9, 2013; test period November 10, 2013 to November 30, 2013.

**Model Robustness Across Countries**: Fig 9 shows the performance of the cascade model for Brazil, Venezuela and Mexico. For Mexico, there are 20 matches out of 21 prediction days which results in 95% accuracy. On the other hand, the cascade model results in 76% accuracy for Venezuela and Brazil. Fig 12 illustrates the ROC plots for each of the countries at various thresholds confirming Brazil and Venezuela’s performance to be worse than Mexico.

### 4.6 Forecasting the Unexpected: The Brazilian Spring

In a recent wave of uprisings in Brazil, known as the Brazilian spring, demonstrations were organized to protest increases in bus, train, and metro ticket prices in some Brazilian cities, which quickly grew to become Brazil’s largest unrest since 1992. These events involved the “General Population.” We test the performance of our cascade-based prediction model by making a retrospective forecast for the events occurred in the month of June 2013 in Brazil. In the training period (November 01, 2012 to May 30, 2013), there were 131 days (out of 212) with events that involved the general population. In the test period of June 2013, there were events almost every day (29 days out of 30). The total number of events was more than 29, since there were multiple events on some days.

For this experiment, we collected 83 million tweets between November 2012 and June 2013 from Brazil. The keyword-based filtering (select if a tweet has at least 3 keywords present) resulted in 890,000 tweets which were further used to generate the graphs and the cascades.

**Fig 13:**
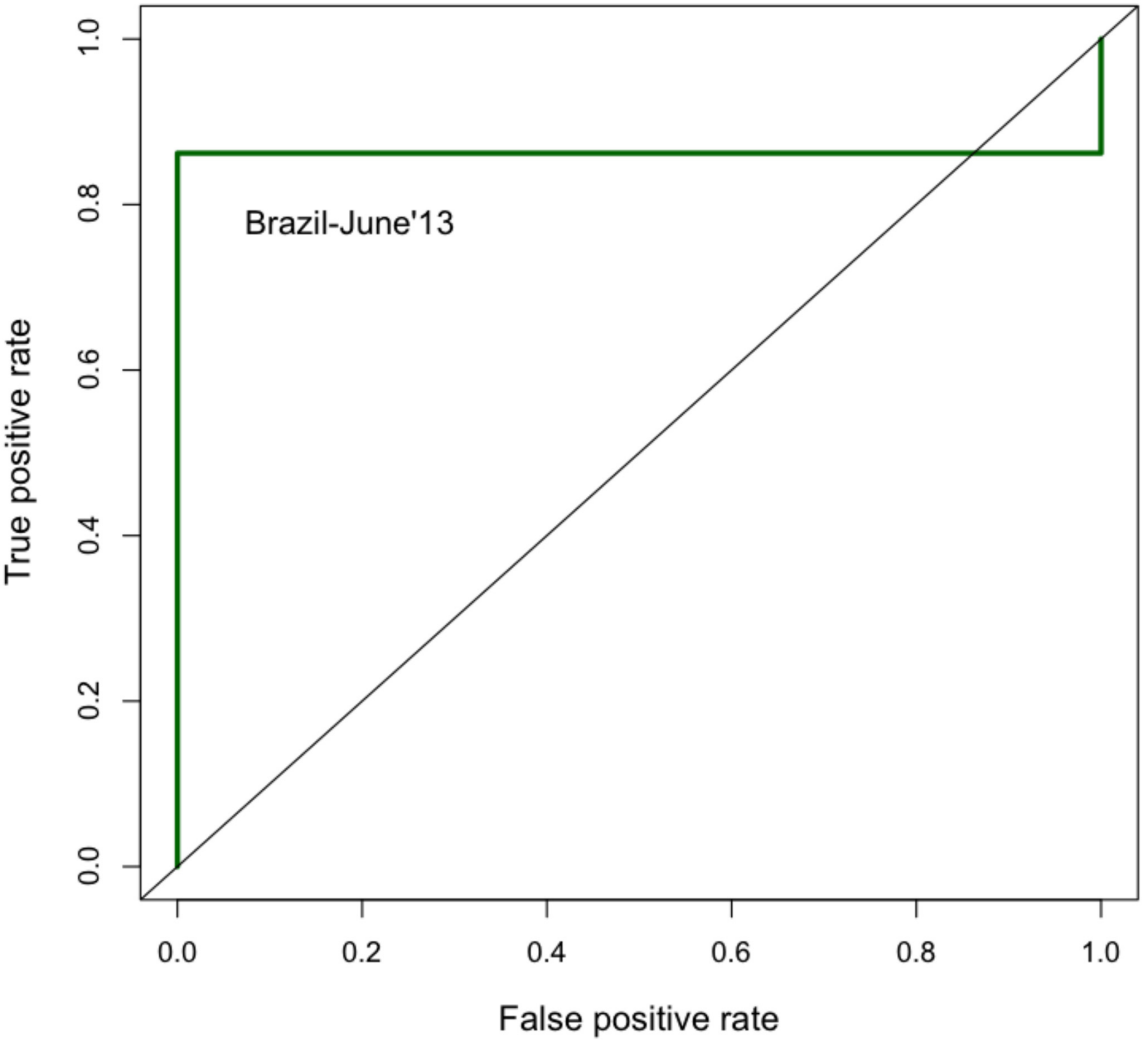
ROC curve for Brazil. ROC curve for different models for Brazil, for a training period of Nov 2012 through May 2013 and testing period of June 1-30, 2013.

**Fig 14:**
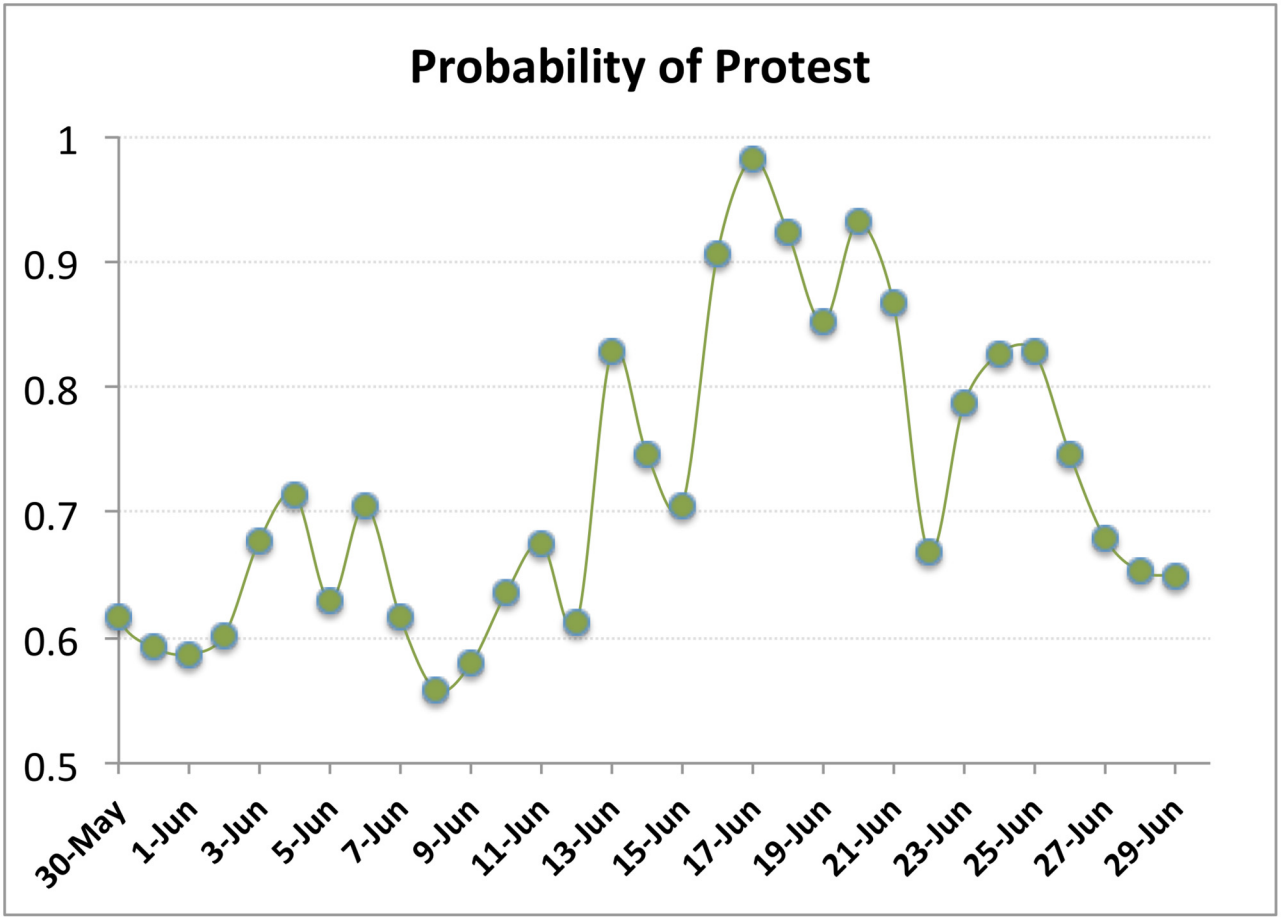
Cascade properties as predictors of protest. Cascade size, number of users, and number of cascades for Follower and MRT cascades in Brazil for the period November 2012—June 2013.

The graph-based features were extracted for each of the cascade-based models. Fig 13 displays the performance of the cascade model for Brazil in June 2013. The model results in an area of 0.86, showing good performance. However, ROC does well when the number of events is very high. Therefore, we also plot the probabilities obtained from the regression model for the test period. Note that the peaks correspond to the days when the events become nation-wide and violent. Fig 14 highlights the sudden surge in the structural features of the cascades. The cascade model results in 25 matches out of 26 alerts (when the best threshold is chosen as 0.6), a performance accuracy of 0.83 and TPR of 0.86.

## Conclusions

Our main contributions include: (i) a detailed analysis of activity cascades arising from protest related tweets, (ii) use of cascade features for a predictive model for protest events, (iii) a rigorous formulation to explain the regimes for small and large cascades, in terms of the spectral radius and the node expansion, and (iv) characterizing critical sets for cascades, by means of the CSSP and CSFP formulations.

Our results suggest that, despite their simplified notion, activity cascades are useful in characterizing and predicting civil unrest events. Our rigorous characterization of the conditions for having large cascades highlights the role of the overall network structure; this corroborates with other recent work on influence cascades [8].

## Supporting Information

S1 Dataset Follower cascade features. (XLS)

S2 Dataset MRT cascade features. (XLS)

S3 Dataset Keyword counts for the volume-based model. (XLS)
